# A Comprehensive Analysis of Gene Expression Evolution Between Humans and Mice

**DOI:** 10.4137/ebo.s2874

**Published:** 2009-07-06

**Authors:** Yupeng Wang, Romdhane Rekaya

**Affiliations:** 1 Department of Animal and Dairy Science; 2 Institute of Bioinformatics; 3 Department of Statistics, University of Georgia Athens, GA 30602, USA. Email:rrekaya@uga.edu

**Keywords:** gene expression, evolution, ortholog, neutral model, promoter

## Abstract

Evolutionary changes in gene expression account for most phenotypic differences between species. Advances in microarray technology have made the systematic study of gene expression evolution possible. In this study, gene expression patterns were compared between human and mouse genomes using two published methods. Specifically, we studied how gene expression evolution was related to GO terms and tried to decode the relationship between promoter evolution and gene expression evolution. The results showed that (1) the significant enrichment of biological processes in orthologs of expression conservation reveals functional significance of gene expression conservation. The more conserved gene expression in some biological processes than is expected in a purely neutral model reveals negative selection on gene expression. However, fast evolving genes mainly support the neutrality of gene expression evolution, and (2) gene expression conservation is positively but only slightly correlated with promoter conservation based on a motif-count score of the promoter alignment. Our results suggest a neutral model with negative selection for gene expression evolution between humans and mice, and promoter evolution could have some effects on gene expression evolution.

## Introduction

Comparative genomics adopts the assumption that important biological processes are often conserved across related species. Based on that, scientists use animal models to infer human physiological and genetic properties.^1^–^3^ Sequence comparison is the most popular tool for comparative genomics. However, sequence similarity is not necessarily proportional to functional similarity.^4^ The biological functions of a gene not only rely on its molecular functions but also its spatiotemporal expression pattern. Changes in gene expression often mean changes in function.^5^ One example is that, for duplicate genes, which are usually associated with highly consistent coding sequences but diverse biological functions, there is only a weak correlation between rates of sequence divergences and rates of expression divergences.^6^ It is urgent to make the details of gene expression evolution clear for the aim of making proper functional inferences across species.

Microarrays, which can characterize the transcriptional profiles of tens of thousands of genes simultaneously, have been widely used in biomedical^7^–^9^ and comparative genomic^10^–^12^ studies. In the latter applications, studies of gene expression levels in different species often rely on cross-species hybridization.^13^–^16^ This method is limited to closely related species as it is based on the hybridization of target RNA and gene probes designed for other species,^17^ and when the probe and target RNA sequences are inconsistent to some extent, this method fails. Even in related species, several studies^18^,^19^ found that this approach may be problematic.

Using microarray data, some theories on gene expression evolution across genomes have been suggested. Yanai et al^20^ found that no expression conservation exists in human and mouse orthologous gene pairs because the evolution of expression profiles of orthologous gene pairs is comparable to that of randomly paired genes. Khaitovich et al^14^ suggested that the majority of expression divergences between species are selectively neutral and are of no functional significance. The above two studies deviated from the idea that genes should be expressed properly to conduct their functions and that basic biological processes are often conserved between related species. Jordan et al^21^ suggested that gene expression divergence among mammalian species is subject to the effects of purifying selective constraint, and it could also be substantially influenced by positive Darwinian selection. Liao and Zhang^22^ found that the expression profile divergence for the majority of orthologous genes between humans and mice is significantly lower than expected under neutrality and is correlated with the coding sequence divergence.

Another issue that should be addressed on the study of gene expression evolution is the relationship between promoter evolution and gene expression evolution. While the premise that the differences in upstream regulatory sequences represent gene expression divergence is widely accepted by researchers, several studies have shown that the changes in transcription factor binding sequences (TFBSs) have only little effect on gene expression evolution.^23^–^26^

The diverse conclusions on gene expression evolution may be due, in part, to the improper comparisons of gene expression patterns across genomes. Expression data should not be compared across probes directly.^22^ Some scientists seek indirect methods, which can make the expression data comparable across probes and even across platforms or species. The conservation of gene co-expression patterns across species has been widely surveyed.^27^–^30^ However, co-expression shows little information on the expression conservation or evolution of orthologous genes across species. To overcome these obstacles, Liao and Zhang^22^ introduced the relative mRNA abundance among tissues (RA) and extracted 26 common tissues between humans and mice to make cross-species expression comparisons possible; Dutilh et al^31^ and Tirosh and Barkai^32^ used either all or most one-to-one orthologs as referred sets for facilitating the gene expression comparisons across genomes.

In this study, we investigated several aspects of gene expression evolution between human and mouse genomes based on olignonucleotide microarray data of humans and mice generated by Su et al,^33^ which is widely used and is one of the largest data sets for humans and mice.^21^,^22^,^34^,^35^ Two methods presented by Liao and Zhang^22^ and Dutilh et al^31^ were adopted and compared for the aim of making reliable conclusions.

## Methods

### Microarray data and orthology

Human and mouse expression data were downloaded from GNF SymAtlas V1.2.4. (http://symatlas.gnf.org/SymAtlas/) by Su et al.^33^ This data set covers 79 human and 61 mouse tissues using the designed Affymetrix microarray chips (human: U133A&GNF1H; mouse: GNF1M). The expression levels were obtained using the MAS 5.0 procedure^36^–^38^ as an average among replicates. To evaluate the reliability of our results, two additional data sets used by Su et al^39^ (retrieved from the Gene Expression Omnibus database at the National Center for Biotechnology Information) and a yeast expression dataset by Spellman et al^40^ (downloaded from http://genome-www.stanford.edu/cellcycle/data/rawdata/) were also analyzed.

The annotation files for GNF1H and GNF1M were downloaded from GNF SymAtlas along with the data files. The annotation file for U133A was downloaded from the Affymetrix website (http://www.affymetrix.com). To assign the Ensembl IDs for each gene, the annotation files (human:uniprot_sprot_human.dat.gz; mouse:uniprot_sprot_rodents.dat.) were downloaded from the Uniprot ftp site at (ftp://us.expasy.org/databases/uniprot/current_release/knowledgebase/taxonomic_divisions/). The orthologous pairs of human and mouse genes and human and yeast genes were downloaded from the Ensembl ftp site (ftp://ftp.ensembl.org/pub/release-47/mysql/compara_mart_homology_47/).

Only one-to-one orthologs were considered for our analyses. The orthologous genes with multiple probe sets were removed from our analyses. The numbers of human and mouse orthologous gene pairs used for this study were 4110 for the dataset by Su et al^33^ and 1960 for the dataset by Su et al.^39^ The number of human and yeast orthologgous gene pairs was 577.

### Comparison of gene expression patterns between genomes

Two procedures presented by Liao and Zhang^22^ (procedure I) and Dutilh et al^31^ (procedure II) were used for comparing gene expression patterns between human and mouse genomes. For procedure I, the expression data of 26 common tissues from two species were extracted and normalized by their relative abundance (RA) values calculated by

$$\begin{array}{c} RA_{H}(i,j)=S_{H}(i,j)/\sum_{j=1}^{n}S_{H}(i,j)\;\;\;\text{and}\\ RA_{M}(i,j)=S_{M}(i,j)/\sum_{j=1}^{n}S_{M}(i,j), \end{array}$$

where *n* is the number of common tissues, *H* represents humans, *M* represents mice, and *S_H_*(*i*, *j*) and *S_M_*(*i*, *j*) are the expression levels of gene *i* in human tissue *j* and mouse tissue *j*, respectively. Then the similarity of gene expression patterns for human and mouse gene *i* is calculated by

$$r_{i}=\frac{\sum_{j=1}^{n}\left[RA_{H}(i,j)RA_{M}(i,j)\right]-\frac{\sum_{j=1}^{n}RA_{H}(i,j)\sum_{j=1}^{n}RA_{M}(i,j)}{n}}{\sqrt{\left(\sum_{j=1}^{n}{\left[RA_{H}(i,j)\right]}^{2}-\frac{{\left[\sum_{j=1}^{n}RA_{H}(i,j)\right]}^{2}}{n}\right)\left(\sum_{j=1}^{n}{\left[RA_{M}(i,j)\right]}^{2}-\frac{{\left[\sum_{j=1}^{n}RA_{M}(i,j)\right]}^{2}}{n}\right)}}.$$

For procedure II, all one-to-one orthologs between humans and mice were used as references. Then the similarity (*r_i_*) of gene expression patterns for human and mouse gene *i* is obtained by correlating the expression correlation values of gene *i* from two different species and the corresponding one-to-one orthologs in their species.

### Calculating Z-scores for GO Terms

For each GO term or gene family, the Z-scores were calculated as:

$$Z=\frac{{\bar{r}}_{s}-{\bar{r}}_{p}}{sd_{p}/\sqrt{n}},$$

where *r̄_s_* is the mean of correlation values of the orthologs in this GO term, *r̄_p_* and *sd_p_* are the mean and standard deviation of correlation values for all available orthologs, and *n* is the number of the available members in this GO term.

### The motif-count score in the alignment of promoter regions

We proposed a motif-count score of the pairwise alignment of promoter sequences, which can be easily derived from the local alignment for two sequences. The promoter sequence was defined as −1000 and +200 bp of the TSS for this study. The matrix for local alignment was constructed with no gap or mismatch allowed. The local alignments with lengths >4 were regarded as conserved DNA motifs/sequences. Each conserved DNA motif/sequence was assigned a score that equaled its length minus 4. The motif-count score for a pairwise alignment of promoter sequences was calculated by summing up the scores of all conserved DNA motifs/sequences in the matrix. Although it is true that some of the conserved DNA motifs/sequences are not true transcription factor binding sites, it is reasonable to assume that the motif-count score based on conserved sequences is generally proportional to that based on true DNA motifs. Thus, the motif-count score allows, in general terms, to measure the similarity of the composition of multiple DNA motifs in two promoter sequences and could help infer biologically the similarity of regulatory patterns for two promoters.

### dN/dS ratio

The nonsynonymous substitution rates (dN), synonymous substitution rates (dS), and their ratio (dN/dS) were used to represent the rates of coding sequence evolution, which were retrieved from the Ensembl ftp web site (ftp://ftp.ensembl.org/pub/release-47/mysql/compara_mart_homology_47/). The ratio of dN/dS is an indicator of selective pressures on coding sequence evolution, where dN/dS > 1 indicates that genes are under positive selection pressure while dN/dS < 1 indicates stabilizing selection.

## Results

### Identification of gene expression conservation in orthologs

By using procedure I and procedure II, the correlations of expression profiles for 4110 human and mouse orthologous genes pairs and random gene pairs were calculated. The results confirmed the theory of non-random expression conservation of orthologs (data not shown), which has been explored by several studies.^22^,^31^,^32^ At the significance level of 1% of genomic background, procedure I and procedure II identified 727 and 559 orthologous gene pairs of expression conservation, which were used for the following functional enrichment analysis of gene expression evolution.

### Analyses of gene expression evolution in terms of biological functions

For orthologous gene pairs that were identified by procedure I and procedure II, we conducted an overrepresented GO term analysis to the human genes using GOstat.^41^ The P value was set at 0.05. The 727 and 559 orthologous gene pairs with expression conservation identified by procedure I and procedure II resulted in 18 and 10 overrepresented terms, respectively (Table 1). The above analysis indicates that the conservation of gene expression has functional significance.

We also investigated whether there are overrepresented GO terms in the human and mouse orthologous genes of fast expression evolution. For that purpose, we retrieved the orthologous genes with the bottom 5% correlation values identified by procedure I and II, respectively. No overrepresented GO terms were returned for these genes. The lack of GO term enrichment in fast evolving genes may be interpreted as evidence for the neutrality of expression evolution. But note that adaptation could involve only few or single genes and does not necessarily require the simultaneous evolution of the expression of the entire GO terms.

**Figure 1:**
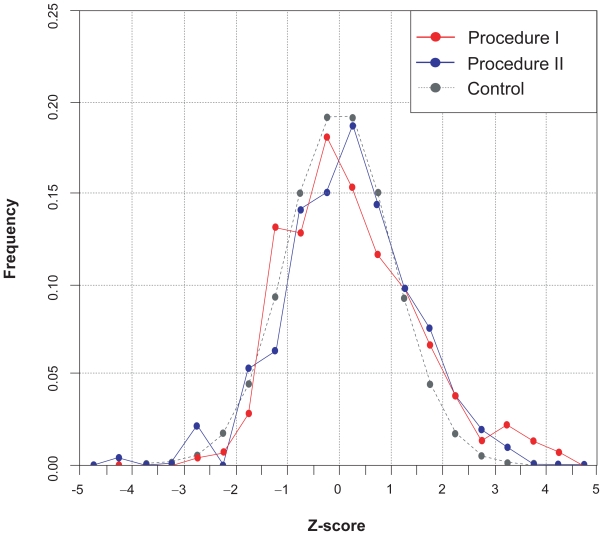
Distribution of Z-scores for GO terms.

To further validate the evolutionary model of gene expression, we investigated how all available GO terms affected gene expression evolution. We took all the orthologous gene pairs as a population and grouped orthologous gene pairs by GO terms. We selected the GO terms with no less than three members and tested 320 terms in all. For each term, we got a Z-score for the mean correlation. Theoretically, these Z-scores should follow a standard normal distribution if no selection exists (note that we removed the GO terms with only one or two members because the means of small size samples may not form the normal distribution if the population does not agree with an exact normal distribution). We plotted the distribution of Z-scores of GO terms against a standard normal distribution (Fig. 1). Generally, the curves formed by procedure I and II fit the neutral model. The distribution of Z-scores for procedure I or II tends to have a heavier right tail (the part of the Z-score >1.96) compared to the control, suggesting that a small part of GO terms have negative selection on gene expression. However, a left heavier tail (the part of the Z-score <−1.96) is not observed, suggesting that generally GO terms do not have obvious positive selection on gene expression.

### Gene expression evolution is slightly correlated with promoter evolution between humans and mice

It is widely accepted by researchers that promoter differences represent regulatory differences, which are reflected by gene expression divergence. However, several studies have indicated that extensively divergent promoters from species may still maintain the same expression patterns,^23^–^25^ which suggests the neutrality of promoter evolution. Zhang et al^42^ found that changes in TFBSs were poorly correlated with divergence of gene expression among yeast paralogs. Tirosh et al^26^ argued that previously identified TFBS of yeasts and mammals had no detectable effect on gene expression. One reason for no detectable or poor correlation may be that an underlying compensatory mechanism allows promoters to rapidly evolve while maintaining a stabilized expression pattern. However, other possibilities should also be considered, e.g. the inherent complexity of promoters, limited data on identified transcription factor binding sites, a suboptimal evolutionary model for promoters, noise of microarray data and improper comparisons of gene expression between species. Thus, it is necessary to reexamine this relationship using new models.

Functional DNA motifs in promoters are often under selection pressure and seem more conserved between species than non functional DNA sequences. Thus, the evolutionary mechanisms of promoters may accommodate different models compared to the model of neutral evolution subject to purifying selection adopted by coding sequences. In addition, it is important to properly designate the similarity between promoters, which will reflect the similarity of gene regulatory patterns besides the sequence similarity. Here we consider three methods for comparing promoter sequences: global alignment, local alignment and our proposed motif-count score method. The global alignment score tends to reflect more the promoter conservation as a whole sequence. The motif-count score of alignments tends to reflect more the conservation of composite DNA motifs by disregarding their positions in the promoters. The local alignment score is somewhat a compromise of the previous two methods.

**Figure 2:**
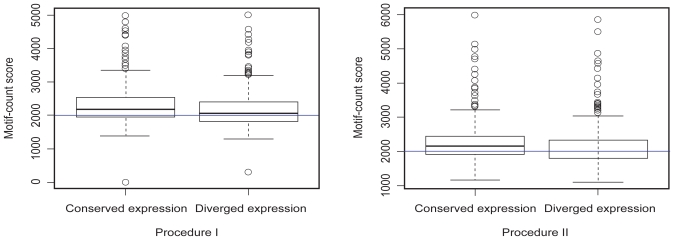
Comparison of the motif-count scores between conserved expression and diverged expression.

Scores based on global alignment, local alignment and motif-count for all orthologs were calculated. Their correlations with gene expression conservations were 0.014 (P value = 0.3772), 0.016 (P value = 0.3087) and 0.055 (P value = 0.0006525), respectively using procedure I; the correlations from procedure II were 0.025 (P value = 0.1218), 0.030 (P value = 0.06608) and 0.040 (P value = 0.01205). With both procedures, the motif-count score method resulted in a slightly positive and significant correlation between promoter conservation and gene expression conservation. The increase of promoter-expression correlation using our proposed motif-count scores suggests it has improved in describing promoter conservation. To reduce the effects of noise in microarray data, we retrieved the most reliable conserved expression (top 10% *r_i_*) and diverged expression (bottom 10% *r_i_*) for analysis. An obvious decrease in motif-count scores from conserved expression to diverged expression is seen in Figure 2 (P values of two sample t test are 0.00289 and 0.003665 for procedure I and II, respectively). The promoter-expression correlations based on these reliable expression patterns were 0.103 (P value = 0.004152) and 0.122 (P value = 0.0006919) using procedures I and II, respectively, indicating a reasonable predictive power of motif-count scores to determine the variability in expression conservation.

From this analysis, it is reasonable to infer that there still could be space for detecting larger promoter-expression correlation if optimal models for describing promoter evolution are used. An optimal model for describing promoter evolution should consider the different evolutionary mechanisms within functional DNA motifs and non functional sequences and the combinational regulatory effects of composite DNA motifs. In this sense, the promoter evolution could indeed affect the gene expression evolution to some extent.

### Reanalyzing gene expression evolution by using other datasets and species

To investigate whether the above conclusions were affected by the choice of the used gene expression dataset, we reanalyzed an additional large microarray dataset used by Su et al.^39^ In total, 1960 pairs of human and mouse orthologs were analyzed. At a significance level of 1% of genomic background, procedures I and II identified 306 and 278 orthologous gene pairs of expression conservation, which confirmed the theory of non-random expression conservation of orthologs. These gene pairs with expression conservation identified by both procedures resulted in 19 and 14 overrepresented GO terms at P value < 0.05, respectively while fast evolving genes had no overrepresented GO terms. The correlations of promoter conservation based on global alignment, local alignment and motif-count scores with gene expression conservation were 0.039 (P value = 0.0967), 0.040 (P value = 0.09138) and 0.058 (P value = 0.01388), respectively using procedure I; 0.034 (P value = 0.1507), 0.040 (P value = 0.08712) and 0.065 (P value = 0.00582), respectively using procedure II.

In addition, we investigated whether these conclusions on gene expression evolution are held when comparing distant species such as humans and yeast. For that purpose, human expression data used by Su et al^33^ and the yeast cell cycle expression data used by Spellman et al^40^ were analyzed. Note that only procedure II can be employed for this analysis. The number of human-yeast one-to-one orthologous pairs was 577. At the significance level of 1% of genomic background, 94 orthologs were identified as having conserved expression, suggesting that the theory of non-random expression conservation of orthologs should be true between humans and yeast. These 94 orthologs returned 11 overrepresented GO terms at P value < 0.05 using GOstat.^41^ Fast evolving genes (bottom 10% *r_i_*) returned no overrepresented GO terms. The above analysis suggests that gene expression conservation has functional significance in both related species and distant species. Finally, we investigated whether gene expression conservation is correlated with promoter conservation. No significant correlation was obtained between *r_i_* and global alignment score (correlation: −0.008, P value = 0.8477), local alignment score (correlation: −0.044, P value = 0.2888) or motif-count score (correlation: −0.067, P value = 0.1056). These results indicate that the weak correlation between promoter conservation and gene expression conservation is not maintained between humans and yeast, which is contrary to the conclusion between humans and mice. The explanation for this finding could be that gene regulatory patterns by DNA motifs may be similar between humans and mice and thus allow their weak correlation with gene expression patterns while gene regulatory patterns are too different between humans and yeast to be correlated with gene expression patterns.

## Discussion

In this study, we analyzed gene expression evolution for orthologs based on human and mouse models. Based on our results, it is reasonable to assume some functional significance for orthologs with expression conservation and neutrality for orthologs with fast expression evolution. Thus, a neutral model with negative selection for gene expression evolution may best explain our results. Additionally, we found a weak correlation between promoter conservation and gene expression conservation. These analyses reveal the inherent complexity of gene expression evolution.

Our neutral model for gene expression evolution differs from previous studies in that the functional significance in gene expression evolution is largely neutral except in some conserved expression patterns; in addition, our model does not mean that gene expression evolution will be well correlated with evolutionary divergence time, evidenced by the fact that determining whether there is a correlation between gene expression divergence and coding sequence divergence is very conflicting in previous studies.^16^,^20^–^22^,^31^,^43^ There could be a possibility that different genes may use different tempos of gene expression evolution with unknown determining factors.

Tirosh et al^26^ tested the changes of DNA motifs in the promoter region to find out if they were correlated with expression divergence and found no detectable correlation. The failure of detecting significant correlations could be due to the limited number of known DNA motifs compared to the unknown true number or/and the lack of proper models for multiple DNA motifs. Zhang et al^42^ used a regression model of multiple DNA motifs to account for gene expression. Although Zhang et al^42^ addressed the promoter-expression correlations based on paralogs while our study was based on orthologs, the conclusions are very similar, suggesting that there could be some mechanisms that promoter evolution affects gene expression evolution.

In this study, the correlations between coding sequence evolution and promoter evolution range from −0.034 to 0.210. Although these correlations may be significant, coding sequence evolution cannot fully account for promoter evolution. Thus, there could be other evolutionary mechanisms in promoters besides nucleotide mutations. We hypothesize that one mechanism may involve mainly the duplication and transposition of DNA motifs, which have been suggested by two previous studies.^44^,^45^ This mechanism may affect gene expression evolution. Our proposed motif-count score reflects some information relative to this mechanism, which may contribute to the detection of promoter-expression association. In addition, two recent studies^46^,^47^ indicated that the evolution of DNA-encoded nucleosome organization and turnover of transcription start sites in promoters may also affect gene expression evolution. We infer that a proper model of promoter evolution considering all mechanisms may be found strongly associated with gene expression evolution.

## Figures and Tables

**Table 1:** The overrepresented GO terms in human and mouse orthologs with expression conservation.

GO term	Description	Number of genes	P value
Procedure I			
GO:0005856	cytoskeleton	52	0.0324
GO:0005624	membrane fraction	43	0.0434
GO:0015629	actin cytoskeleton	18	0.0434
GO:0005509	calcium ion binding	53	0.0434
GO:0004867	serine-type endopeptidase inhibitor activity	11	0.0434
GO:0044430	cytoskeletal part	34	0.0434
GO:0000267	cell fraction	58	0.0434
GO:0016052	carbohydrate catabolic process	15	0.0434
GO:0009605	response to external stimulus	52	0.0434
GO:0006936	muscle contraction	18	0.0434
GO:0007286	spermatid development	7	0.0434
GO:0016491	oxidoreductase activity	54	0.0434
GO:0006941	striated muscle contraction	6	0.0434
GO:0006006	glucose metabolic process	15	0.0434
GO:0008236	serine-type peptidase activity	18	0.0434
GO:0019318	hexose metabolic process	18	0.0434
GO:0019320	hexose catabolic process	11	0.0445
GO:0048232	male gamete generation	21	0.0469
Procedure II			
GO:0048232	male gamete generation	21	0.000537
GO:0043232	intracellular non-membrane-bounded organelle	74	0.000537
GO:0019953	sexual reproduction	25	0.000684
GO:0006996	organelle organization	52	0.00322
GO:0007276	gamete generation	22	0.00433
GO:0007286	spermatid development	7	0.00913
GO:0051276	chromosome organization	22	0.00917
GO:0006323	DNA packaging	19	0.0161
GO:0016585	chromatin remodeling complex	7	0.02
GO:0016043	cellular component organization	101	0.0464
